# Hydroperoxyl Radical Scavenging Activity of Bromophenols from Marine Red Alga *Polysiphonia urceolata*: Mechanistic Insights, Kinetic Analysis, and Influence of Physiological Media

**DOI:** 10.3390/molecules30081697

**Published:** 2025-04-10

**Authors:** Houssem Boulebd

**Affiliations:** Laboratory of Synthesis of Molecules with Biological Interest, Department of Chemistry, Faculty of Exact Sciences, University of Frères Mentouri Constantine 1, Constantine 25017, Algeria; boulebd.houssem@umc.edu.dz

**Keywords:** marine natural products, bromophenols, hydroperoxyl radical scavenging, DFT calculations, kinetic analysis

## Abstract

Bromophenols (BPs), particularly those derived from marine sources, are known for their potent radical scavenging activity, effectively neutralizing reactive oxygen species (ROS). However, their exact mechanism of action remains largely unexplored, limiting our understanding of their potential as natural antioxidants. In this study, the antiradical mechanisms of two BP derivatives (**1** and **2**), previously isolated from the marine red alga *Polysiphonia urceolata*, were systematically investigated using thermodynamic and kinetic calculations. Both compounds demonstrated potent hydroperoxyl radical (HOO^•^) scavenging activity in polar and lipid environments, with rate constants surpassing those of the well-known antioxidant standards Trolox and BHT. In lipid media, BP **2** exhibited approximately 600-fold greater activity than BP **1**, with rate constants of 9.75 × 10^5^ and 1.64 × 10^3^ M^−1^ s^−1^, respectively. In contrast, both BPs showed comparable activity in aqueous media, with rate constants of 3.46 × 10^8^ and 9.67 × 10^8^ M^−1^ s^−1^ for **1** and **2**, respectively. Mechanistic analysis revealed that formal hydrogen atom transfer (*f*-HAT) is the predominant pathway for radical scavenging in both lipid and polar environments. These findings provide critical insights into the antiradical mechanisms of natural BPs and underscore the potential of BP **1** and BP **2** as highly effective hydroperoxyl radical scavengers under physiological conditions.

## 1. Introduction

Bromophenols (BPs) derived from marine algae exhibit a wide range of biological activities, making them promising candidates for pharmaceutical applications [1,2]. Several studies have demonstrated their potent anticancer properties, with compounds isolated from *Leathesia nana* and *Rhodomela confervoides* showing cytotoxic effects against various human cancer cell lines, including A549 (lung carcinoma), HCT-116 (colon carcinoma), and HeLa (cervical carcinoma) [3]. In addition to their anticancer effects, BPs possess antimicrobial activity, particularly against *Candida albicans* and *Porphyromonas gingivalis* [4,5]. Some BPs also demonstrate antifungal properties, inhibiting phytopathogenic fungi such as *Botrytis cinerea* and *Fusarium graminearum* [6]. Moreover, certain BPs show antiviral potential, with activity against fish pathogenic viruses like infectious hematopoietic necrosis virus (IHNV) and infectious pancreatic necrosis virus (IPNV) [7]. Beyond their antimicrobial action, BPs have been investigated for their anti-inflammatory potential. Studies suggest that they alleviate IgE-mediated inflammation, suppress NF-κB and STAT1 signaling pathways, and reduce the production of pro-inflammatory cytokines such as IL-6 and TNF-α [8]. Furthermore, BPs have been identified as neuroprotective agents with potential applications in neurodegenerative diseases such as Alzheimer’s and Parkinson’s disease [9]. By inhibiting key enzymes like acetylcholinesterase (AChE), butyrylcholinesterase (BChE), and glycogen synthase kinase-3β (GSK-3β), they help prevent the formation of β-amyloid plaques, a hallmark of Alzheimer’s disease [10].

**Figure 1:**
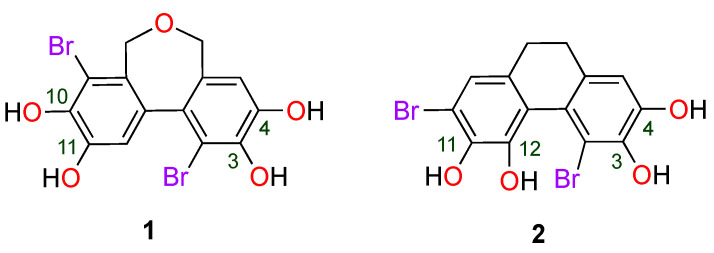
Molecular structure of the investigated bromophenols.

Due to their unique chemical structures BPs have been reported to exhibit significant antioxidant activity [11]. They are effective in scavenging free radicals such as DPPH and ABTS, with IC_50_ values often in the micromolar range, indicating high potency [2]. Mechanistically, the antioxidant activity of BPs is attributed to their ability to donate hydrogen atoms or electrons, which is facilitated by the presence of bromine and hydroxyl groups on their aromatic rings [12]. This structural configuration enhances radical scavenging and prevents oxidative damage in biological systems. Among natural BPs, 1,8-dibromo-5,7-dihydrodibenzo [c,e] oxepine-2,3,9,10-tetraol (**1**) and 4,7-dibromo-9,10-dihydrophenanthrene-2,3,5,6-tetraol (**2**) (Figure 1) have demonstrated excellent antiradical activity in vitro [13]. These compounds were isolated from the marine red alga *Polysiphonia urceolata*, and their antiradical activity was evaluated using the DPPH assay, with comparisons to the standard antioxidant BHT (butylated hydroxytoluene). Both compounds exhibited significantly higher DPPH scavenging activity than BHT, with IC_50_ values in the range 6–8 µM, compared to 83.8 µM for BHT. This highlights the potent antiradical activity of these natural compounds. From a structural point of view, BPs **1** and **2** share common features: they both consist of two aromatic rings linked by a single bond. Each aromatic ring bears a bromine atom and two adjacent hydroxyl (OH) groups. Additionally, a second bond between the rings forms a third ring, which is six-membered in compound **1** and seven-membered in compound **2**. The primary difference between these two structures lies in the nature of the ring connecting the two aromatic cores: in compound **1**, this ring contains an oxygen atom, which could influence its electronic properties and chemical reactivity.

As part of our ongoing efforts to elucidate the mechanisms underlying the antioxidant activity of natural compounds [14,15], this study provides a comprehensive examination of the antiradical mechanisms of **1** and **2**, selected as representative derivatives of naturally occurring BPs. The aim of this investigation is to provide a detailed understanding of the mode of action of these compounds and the influence of their structural features on antiradical activity. To achieve this goal, we employed a robust methodology based on density functional theory (DFT) calculations that accounts for all potential reaction mechanisms at all the possible reactive sites of the molecules. Additionally, the influence of environmental factors, such as pH and solvent polarity, was systematically evaluated to provide a complete picture of their antioxidant behavior under physiologically relevant conditions.

## 2. Results and Discussion

### 2.1. Molecular Geometry and Electronic Properties

**Figure 2:**
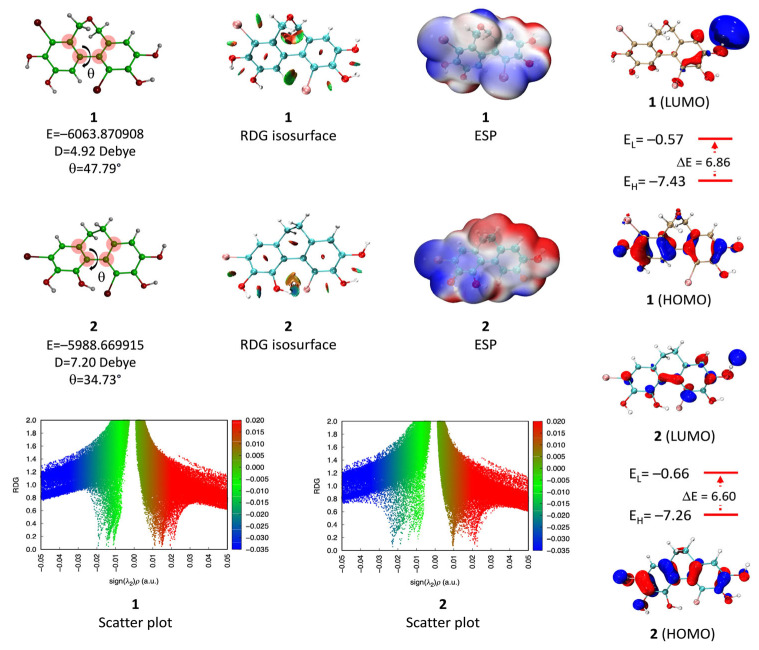
Computed optimized molecular geometries, RDG isosurfaces, Scatter plots, and frontier molecular orbitals (FMOs) of BPs **1** and **2**, calculated at the M06-2X/6-311++G(d, p) level.

The molecular geometry and electronic properties of BPs **1** and **2** have been determined at the theoretical level M06-2X/6-311++G(d, p). Figure 2 shows the optimized structures of the two BPs, along with their energies, dipole moments, frontier molecular orbital (HOMO and LUMO), electrostatic potential (ESP), and non-covalent interactions represented by RDG isosurfaces and Scatter plots. Analysis of dipole moments reveals that BP **2** (7.20 Debye) is more polar than BP **1** (4.92 Debye). This difference can be attributed to the presence of the oxygen atom in **1**, which favors a more homogeneous distribution of electronic charge, thus reducing the asymmetry of charge distribution. The electrostatic potential (ESP) study confirms this trend, highlighting a more uniform distribution of electronic charge in BP **2** than in BP **1**. In both cases, electron-deficient regions (in red on the ESP) are mainly located on the hydrogens of phenolic groups, while electron-rich areas (in blue) are mainly located on bromine and oxygen atoms.

The RDG isosurfaces identify non-covalent interactions present in molecules. The green areas in the figure are characteristic of Van der Waals interactions, while the red and blue regions reveal attractive (hydrogen bonds) and repulsive (steric effects) interactions, respectively. Furthermore, the dispersion plots sign (λ2) ρ vs. RDG allow quantitative evaluation of these interactions. For both BPs, several attractive interactions of the Van der Waals type, as well as hydrogen bonds, are observed, notably within the catechol motif and between the phenolic hydroxyl groups and the bromine atoms. These interactions contribute significantly to the stability of the molecules. The analysis of the frontier molecular orbitals shows that the HOMO and LUMO energies vary slightly between the two compounds. For BP **1**, the HOMO energy (E_H_ = −7.43 eV) and the LUMO energy (E_L_ = −0.57 eV) give an energy gap ΔE = 6.86 eV. In contrast, for BP **2**, the values are E_H_ = −7.26 eV and E_L_ = −0.66 eV, with a ΔE = 6.60 eV gap. A lower HOMO–LUMO gap for BP **2** suggests higher chemical reactivity.

### 2.2. Radical Scavenging Mechanism at Physiological Conditions

#### 2.2.1. Acid–Base Equilibrium

**Figure 3:**
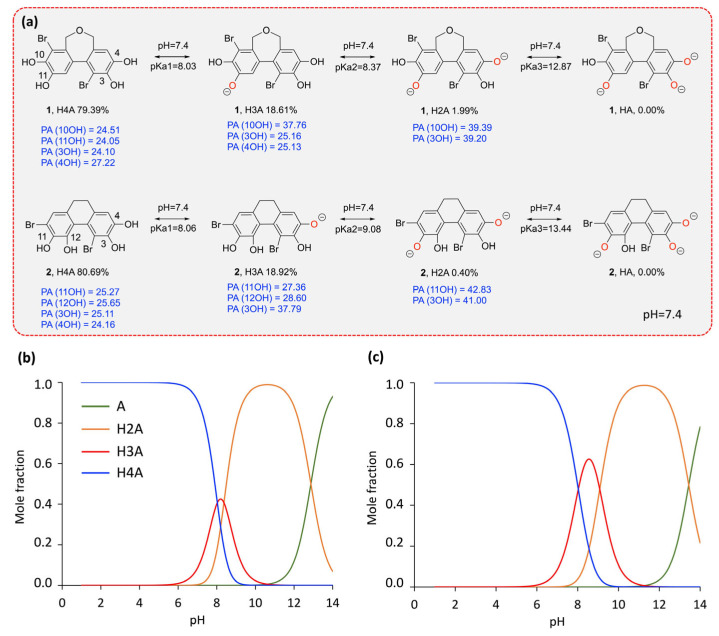
Computed pKa values and acid–base equilibrium of BPs **1** and **2** at physiological pH (**a**), and molar fraction of neutral and deprotonated forms as a function of pH for BP **1** (**b**) and BP **2** (**c**).

It is well established that the antioxidant activity of phenolic compounds is largely influenced by the acid–base equilibrium in water [16,17,18]. Depending on the pKa value, a phenolic compound can deprotonate spontaneously at physiological pH, leading to the formation of an anionic species able to neutralize free radicals via hydrogen transfer (HAT) or electron transfer (SET) mechanisms. Consequently, the deprotonation of the studied BPs at physiological pH must be taken into account in aqueous environments. This section therefore evaluates the behavior of BPs **1** and **2** at physiological pH, as well as over a pH range from 1 to 14. The calculated pKa values are presented in Figure 3, along with the variation of the mole fraction of neutral and deprotonated species as a function of pH and at physiological pH.

For BP **1**, the pKa values obtained are 8.30, 8.37, and 12.87, corresponding to the neutral (H4A), monodeprotonated (H3A), and doubly deprotonated (H2A) forms, respectively. Similarly, BP **2** has pKa values of 8.06, 9.08, and 13.44, following the same protonation sequence. At physiological pH (7.4), the molar fractions of these species indicate a dominance of the neutral form, with BP **1** existing at 79.39% in neutral form, 18.61% in monodeprotonated form, and 1.99% in doubly deprotonated form. BP **2**, on the other hand, shows a similar but slightly different distribution: 80.69% in neutral form, 18.92% in monodeprotonated form, and only 0.4% in doubly deprotonated form. These proportions suggest that both compounds remain predominantly neutral at physiological pH, with a significant fraction in the monodeprotonated form and a small proportion in the doubly deprotonated form. Based on these results, both the neutral and deprotonated forms were investigated in water, while only the neutral form was considered under lipid conditions.

#### 2.2.2. Thermodynamic Assessment in Physiological Media

**Figure 4:**
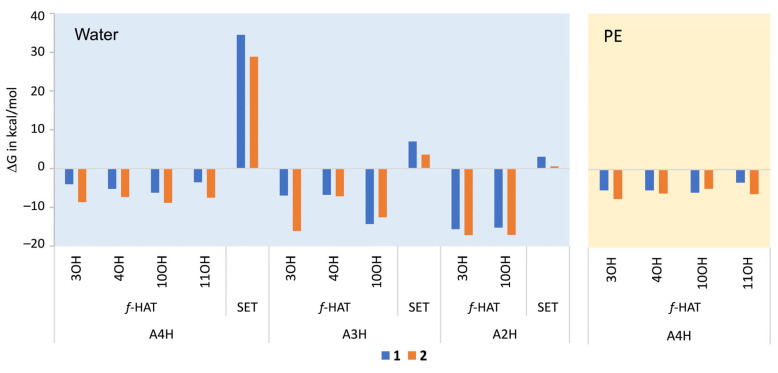
Computed ΔG° values of the reactions of BPs **1** and **2** with HOO^•^ radical following *f*-HAT and SET mechanisms in physiological media.

A thermodynamic evaluation in physiological media was performed for BP **1** and **2** to assess their efficiency in scavenging HOO^•^ radicals via the formal hydrogen atom transfer (*f*-HAT) and single-electron transfer (SET) mechanisms at all potential reaction sites. The computed Gibbs free energy changes (ΔG°) are presented in Figure 4. The *f*-HAT mechanism was found to be thermodynamically favorable at all hydroxyl groups in both polar and lipid media, with ΔG° values ranging from −3.54 to −17.18 kcal/mol. Comparatively, the ΔG° values for the *f*-HAT pathway in both media are slightly lower for BP 2 than for BP **1**, suggesting that BP **2** may exhibit higher reactivity toward free radicals. For both BPs, it was also observed that *f*-HAT from the deprotonated catechol moieties exhibits the lowest ΔG° values (ranging from −14.28 to −15.63 kcal/mol for BP **1** and from −16.10 to −17.18 kcal/mol for BP **2**), indicating that these sites are the most favorable for hydrogen transfer. This finding is consistent with previous studies conducted on catechol-containing antioxidants [19].

For the SET mechanism, electron transfer is more energetically favorable from deprotonated forms (ΔG° = 3.09–7.01 kcal/mol) than from neutral forms (ΔG° = 28.84–34.49 kcal/mol). However, all reactions are characterized by positive ΔG° values, indicating that these processes are endergonic. Despite this energy requirement, the SET mechanism involving deprotonated forms was considered in the following kinetic studies due to the rapidity with which these reactions can occur. This apparent contradiction between thermodynamics and kinetics is explained by the fact that, even though the Gibbs free energy change is positive, SET reactions can be strongly favored by kinetic factors, such as the stabilization of reaction intermediates or a low activation barrier.

#### 2.2.3. Kinetic Investigations in Physiological Media

**Figure 5:**
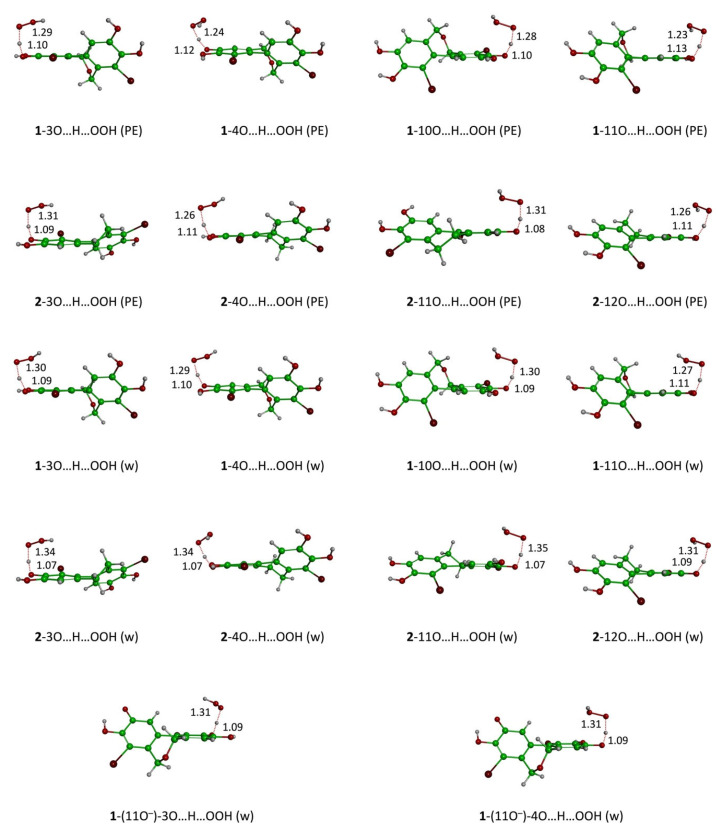
Localized TSs of the reaction of BPs **1** and **2** with the HOO^•^ radical following f-HAT mechanisms in physiological media.

The reaction kinetics of BPs **1** and **2** in physiological media were investigated using the QM-ORSA protocol [19,20]. The overall rate constants were determined using the following equations, and the results are summarized in Table 1 and Table 2. The corresponding transition states are depicted in Figure 5. Notably, for some *f*-HAT processes, the transition states could not be localized, indicating that these processes are barrierless and occur at diffusion-controlled rates. This behavior is particularly evident in *f*-HAT processes involving deprotonated catechol moieties.

In pentyl ethanoate:

$$k_{overall}=\Sigma k_{app}\left(fHAT\right)$$

In water:

$$k_{overall}=\Sigma k_{f}\left(fHAT\right)+\Sigma k_{f}\left(SET\right)$$

The calculated *k*_overall_ values in pentyl ethanoate for BPs **1** and **2** were determined to be 1.64 × 10^3^ and 9.75 × 10^5^ M^−1^ s^−1^, respectively. These results indicate that BP **2** is approximately 600 times more reactive toward the HOO^•^ radical than BP **1**. This significant difference in reactivity suggests that the presence of the oxygen atom in BP **1** negatively impacts its antiradical activity compared to BP **2**. This observation is in line with the results from the DPPH assay conducted by Li et al. [13], which demonstrated that the IC_50_ of BP **1** is higher than that of BP **2** (6.1 mM vs. 8.1 mM). Both findings consistently highlight that BP **2** exhibits superior antiradical performance compared to BP **1**.

Analysis of the reaction sites revealed that hydrogen abstraction predominantly occurs at the 3-OH group, with branching ratios of 83% for BP **1** and 93% for BP **2**. When compared to other well-known antioxidants, the overall rate constant of BP **2** (9.75 × 10^5^ M^−1^ s^−1^) surpasses those of Trolox (*k* = 3.40 × 10^3^ M^−1^ s^−1^) [21], BHT (*k*_overall_ = 1.70 × 10^4^ M^−1^ s^−1^) [22], psoralidin (*k*_overall_ = 1.21 × 10^4^ M^−1^ s^−1^) [15], feruloylquinic acid (*k*_overall_ = 4.10 × 10^4^ M^−1^ s^−1^) [18], and cannabidiolic acid (*k*_overall_ = 1.36 × 10^3^ M^−1^ s^−1^) [23]. This highlights the potent HOO^•^ scavenging activity of BP **2** in lipid media. In contrast, BP **1** exhibits moderate to good radical scavenging activity.

Regarding reactivity in the aqueous environment, the overall rate constants of the two BPs exhibit values of the same order of magnitude, with the *k*_overall_ value for BP **1** being approximately three times lower than that of BP **2** (3.46 × 10^8^ vs. 9.67 × 10^8^ M^−1^ s^−1^, respectively). This suggests that BP **2** is slightly more reactive than BP **1**. Nevertheless, both compounds demonstrate significantly higher reactivity in water compared to BHT (*k*_overall_ = 2.51 × 10^5^ M^−1^ s^−1^) [22], which is fully consistent with the results obtained from the DPPH assay. Furthermore, the rate constants of both BPs are also higher than those of known antioxidants such as ascorbic acid (*k* = 9.97 × 10^7^ M^−1^ s^−1^) [19], Trolox (*k* = 8.96 × 10^4^ M^−1^ s^−1^) [21], and cannabidiolic acid (*k*_overall_ = 2.40 × 10^6^ M^−1^ s^−1^) [23], confirming their effectiveness as inhibitors of peroxyl radicals under physiological polar conditions. The monodeprotonated form (H3A) of both molecules contributes predominantly to the rate constant, accounting for 81% for BP **1** and 97% for BP **2**. The *f*-HAT mechanism is dominant for both compounds. For BP **1**, this mechanism occurs selectively at the hydroxyl group in the 10-position (10-OH), while for BP **2**, it can occur equivalently on the three non-deprotonated hydroxyl groups; 3-OH, 11-OH, and 12-OH. Regarding the SET mechanism, the specific rate constants associated with this pathway are relatively high, ranging from 10^5^ to 10^7^ M^−1^ s^−1^ for both compounds, indicating a rapid process. However, compared to the values observed for the *f*-HAT mechanism, these contributions remain negligible. Thus, the *f*-HAT mechanism is the primary process in the aqueous environment at physiological pH.

## 3. Materials and Methods

All density functional theory (DFT) computations were carried out using the Gaussian 09 software [24]. The M06-2X hybrid meta-exchange-correlation functional, combined with the 6-311++G(d, p) basis set, was employed, as it has been validated for similar systems, providing reliable results in agreement with experimental data [25,26,27]. To model physiological environments, Truhlar’s SMD solvation model was utilized, with water and pentyl ethanoate serving as solvents to represent polar and lipid conditions, respectively [28]. Transition states (TSs) and ground states were identified through imaginary frequency (IF) analysis, and TSs were further validated using intrinsic reaction coordinate (IRC) calculations. For post-processing analyses, Multiwfn 3.8 software was used to compute frontier molecular orbitals (FMOs), electrostatic potential maps (ESPs), and noncovalent interactions based on the reduced density gradient (NCI-RDG) [29]. Visualization of these properties was performed using VMD 1.9.3 [30]. The pKa values were calculated according to a protocol described in the literature [31]. The cartesian coordinates of the localized TSs of the reaction between BPs **1** and **2** and HOO^•^ in physiological media are reported in Table S1 in the Supplementary Materials.

Kinetic parameters were derived using the quantum-mechanics-based test for overall free radical scavenging activity (QM-ORSA) methodology [19,20]. The rate constant (*k*) was calculated using standard transition state theory (TST) at 298.15 K with a 1 M standard state, as follows [32,33,34]:

$$k=\sigma \kappa \frac{k_{B}T}{h}e^{-({\Delta G}^{\neq })/RT}$$

where *σ* is the reaction symmetry number [35,36], *κ* represents tunneling corrections computed using the Eckart barrier [37], *k_B_* is the Boltzmann constant, *h* is the Planck constant, and ∆*G*^≠^ is the Gibbs free energy of activation.

For reactions approaching the diffusion limit, apparent rate constants were adjusted using the Collins–Kimball theory [38]. Branching ratios (Γ, %) were determined using:

$$\Gamma_{path}=\frac{k}{k_{overall}}\times 100$$

where *k* is the rate constant for a specific reaction pathway and *k*_overall_ is the total rate constant of all pathways.

The Gibbs free energy of activation for the single-electron transfer (SET) mechanism was estimated using Marcus theory [39]:

$${∆G}_{SET}^{\neq }=\frac{\lambda }{4}{\left(1+\frac{{∆G}_{SET}^{0}}{\lambda }\right)}^{2}$$

$$\lambda \approx {\Delta E}_{SET}+{∆G}_{SET}^{0}$$

where λ represents the nuclear reorganization energy, ${∆G}_{SET}^{0}$ is the Gibbs free energy of reaction, and ${\Delta E}_{SET}$ is the nonadiabatic energy difference between the reactants and vertical products.

## 4. Conclusions

The hydroperoxyl radical scavenging activity and mechanisms of BPs **1** and **2**, previously isolated from *Polysiphonia urceolata*, have been thoroughly investigated using DFT calculations and kinetic studies. The results demonstrate that both compounds exhibit good to high scavenging capacity in lipid media, with BP **2** emerging as the most active derivative. Notably, the rate constant of BP **2** in lipid media was found to exceed that of common antioxidants such as Trolox and BHT. In aqueous media at physiological pH, both compounds exhibit comparable reactivity, with rate constants in the order of 10^8^ M^−1^ s^−1^, yet they are significantly more active than both BHT and Trolox. Mechanistically, the *f*-HAT pathway was identified as the primary mechanism in both lipid and aqueous media for both BPs. In lipid media, *f*-HAT occurs at the 3-OH site, while in water, it takes place at the 10-OH site for BP **1** and at the 3-OH, 11-OH, and 12-OH sites for BP **2**. The findings obtained are in good agreement with the results of the DPPH assay reported in the literature, which confirms the accuracy of the calculations. This study provides new insights into the mechanisms and antioxidant capacity of BPs and highlihts **1** and **2** as potent natural antioxidants in both polar and lipid environments.

## Figures and Tables

**Table 1:** Kinetic data of the reactions HOO^•^ radicals with BP **1** and **2** in pentyl ethanoate.

Compound	Position	IF ^a^ (cm^−1^)	ΔG^≠ b^ (kcal/mol)	κ ^c^	*k* ^d^ (M^−1^ s^−1^)	Γ ^e^ (%)	*k*_overall_ (M^−1^ s^−1^)
**1**	3OH	−2026.7	15.4	44.7	1.35 × 10^3^	83	1.64 × 10^3^
	4OH	−2440.8	17.8	360.9	1.78 × 10^2^	11	
	10OH	−2040.1	18.1	109.4	3.70 × 10^1^	2	
	11OH	−2434.1	18.4	401.6	7.02 × 10^1^	4	
**2**	3OH	−1880.6	11.9	9.7	9.75 × 10^4^	93	9.75 × 10^5^
	4OH	−2467.8	15.4	127.6	3.91 × 10^3^	4	
	10OH	−1901.2	15.93	39.7	5.18 × 10^2^	0	
	11OH	−2307.9	15.42	102.2	3.16 × 10^3^	3	

^a^ imaginary frequency, ^b^ activation free energy, ^c^ tunneling correction, ^d^ rate constant, and ^e^ branching ratio.

**Table 2:** Kinetic data of the reactions of HOO^•^ radicals with BP **1** and **2** in water at pH = 7.4.

Comp.	Mechanism		State	ΔG^≠ a^ (kcal/mol)	κ ^b^	*k*_app_ ^c^ (M^−1^ s^−1^)	*f* ^d^	*k*_f_ ^e^ (M^−1^ s^−1^)	Γ ^f^ (%)	*k*_overall_ (M^−1^ s^−1^)
**1**	*f*-HAT	3OH	H4A	17.33	252.31	3.09 × 10^2^	0.794	2.45 × 10^2^	0	3.46 × 10^8^
		4OH		19.72	1184.32	2.57 × 10^1^		2.04 × 10^1^	0	
		10OH		17.97	650.68	2.70 × 10^2^		2.14 × 10^2^	0	
		11OH		20.29	1361.70	1.13 × 10^1^		8.97 × 10^0^	0	
	*f*-HAT	3OH	H3A	16.16	166.10	1.47 × 10^3^	0.186	2.73 × 10^2^	0	
		4OH		17.99	545.21	2.20 × 10^2^		4.09 × 10^1^	0	
		10OH		-	-	1.50 × 10^9 g^		2.79 × 10^8^	81	
	SET			5.74	16.25 ^h^	4.73 × 10^6^		8.80 × 10^5^	0	
	*f*-HAT	3OH	H2A	-	-	1.50 × 10^9 g^	0.020	3.00 × 10^7^	9	
		10OH		-	-	1.50 × 10^9 g^		3.70 × 10^7^	9	
	SET			8.34	16.46 ^h^	3.30 × 10^8^		6.60 × 10^6^	2	
**2**	*f*-HAT	3OH	H4A	12.24	24.48	1.62 × 10^5^	0.807	1.31 × 10^5^	0	9.67 × 10^8^
		4OH		14.96	205.97	1.39 × 10^4^		1.12 × 10^4^	0	
		11OH		13.34	72.39	7.51 × 10^4^		6.06 × 10^4^	0	
		12OH		15.47	407.47	1.14 × 10^4^		9.20 × 10^3^	0	
	*f*-HAT	3OH	H3A	-	-	1.60 × 10^9 g^	0.189	3.02 × 10^8^	31	
		11OH		-	-	1.60 × 10^9 g^		3.02 × 10^8^	31	
		12OH		-	-	1.60 × 10^9 g^		3.02 × 10^8^	31	
	SET			6.09	16.28 ^h^	1.97 × 10^8^		3.72 × 10^7^	4	
	*f*-HAT	3OH	H2A	-	-	1.60 × 10^9 g^	0.004	6.40 × 10^6^	1	
		12OH		-	-	1.60 × 10^9 g^		6.50 × 10^6^	1	
	SET			2.20	13.18 ^h^	2.35 × 10^9^		9.40 × 10^6^	1	

^a^ activation free energy, ^b^ tunneling correction, ^c^ apparent rate constant, ^d^ mole fraction, ^e^ *k*_f_ = *f*·*k*_app_, ^f^ branching ratio, ^g^ diffusion rate constant, ^h^ the nuclear reorganization energy (λ).

## Data Availability

The data supporting the findings of this study are available in the article and its Supplementary Materials.

## Supplementary Materials

The following supporting information can be downloaded at: https://www.mdpi.com/article/10.3390/molecules30081697/s1, Table S1. The Cartesian coordinates of the TSs of the reaction between BPs **1** and **2** and HOO^•^ radicals following the *f*-HAT mechanism in physiological media at the M06-2X/6-311++G(d, p) level.

## References

[1] Dong H., Hansen P.E., Dong S., Stagos D., Lin X., Liu M., Pérez-Correa J.R., Mateos R., Domínguez H. (2023). 3—Marine natural bromophenols: Sources, structures, main bioactivities, and toxicity. Marine Phenolic Compounds.

[2] Dong H., Dong S., Erik Hansen P., Stagos D., Lin X., Liu M. (2020). Progress of Bromophenols in Marine Algae from 2011 to 2020: Structure, Bioactivities, and Applications. Mar. Drugs.

[3] Matulja D., Vranješević F., Kolympadi Markovic M., Pavelić S.K., Marković D. (2022). Anticancer Activities of Marine-Derived Phenolic Compounds and Their Derivatives. Molecules.

[4] Xu X., Yin L., Wang Y., Wang S., Song F. (2013). A new bromobenzyl methyl sulphoxide from marine red alga Symphyocladia latiuscula. Nat. Prod. Res..

[5] Cherian C., Jannet Vennila J., Sharan L. (2019). Marine bromophenols as an effective inhibitor of virulent proteins (peptidyl arginine deiminase, gingipain R and hemagglutinin A) in Porphyromas gingivalis. Arch. Oral Biol..

[6] Liu M., Wang G., Xiao L., Xu X., Liu X., Xu P., Lin X. (2014). Bis(2,3-dibromo-4,5-dihydroxybenzyl) Ether, a Marine Algae Derived Bromophenol, Inhibits the Growth of Botrytis cinerea and Interacts with DNA Molecules. Mar. Drugs.

[7] Kim S.-Y., Kim S.R., Oh M.-J., Jung S.-J., Kang S.Y. (2011). In Vitro antiviral activity of red alga, Polysiphonia morrowii extract and its bromophenols against fish pathogenic infectious hematopoietic necrosis virus and infectious pancreatic necrosis virus. J. Microbiol..

[8] Kang N.-J., Han S.-C., Kang H.-J., Ko G., Yoon W.-J., Kang H.-K., Yoo E.-S. (2017). Anti-Inflammatory Effect of 3-Bromo-4,5-Dihydroxybenzaldehyde, a Component of Polysiphonia morrowii, In Vivo and In Vitro. Toxicol. Res..

[9] Paudel P., Park S.E., Seong S.H., Jung H.A., Choi J.S. (2020). Bromophenols from Symphyocladia latiuscula Target Human Monoamine Oxidase and Dopaminergic Receptors for the Management of Neurodegenerative Diseases. J. Agric. Food Chem..

[10] Paudel P., Seong S.H., Zhou Y., Park H.J., Jung H.A., Choi J.S. (2019). Anti-Alzheimer’s Disease Activity of Bromophenols from a Red Alga, Symphyocladia latiuscula (Harvey) Yamada. ACS Omega.

[11] Tziveleka L.-A., Tammam M.A., Tzakou O., Roussis V., Ioannou E. (2021). Metabolites with Antioxidant Activity from Marine Macroalgae. Antioxidants.

[12] Boulebd H. (2024). Mechanistic Insights into the Antioxidant and Pro-oxidant Activities of Bromophenols from Marine Algae: A DFT Investigation. J. Org. Chem..

[13] Li K., Li X.-M., Ji N.-Y., Wang B.-G. (2008). Bromophenols from the Marine Red Alga Polysiphonia urceolata with DPPH Radical Scavenging Activity. J. Nat. Prod..

[14] Boulebd H., Amine Khodja I., Benarous K., Mą̨czyński M., Spiegel M. (2025). A Comprehensive Experimental and Theoretical Investigation of the Antioxidant Properties of Hispidin and Isohispidin. J. Org. Chem..

[15] Boulebd H., Pereira D.M. (2023). Examination of the Antioxidant Activity of Psoralidin: Computational Mechanistic Study and Impact on the ROS Level in Human Keratinocytes. J. Org. Chem..

[16] Amorati R., Pedulli G.F., Cabrini L., Zambonin L., Landi L. (2006). Solvent and pH Effects on the Antioxidant Activity of Caffeic and Other Phenolic Acids. J. Agric. Food Chem..

[17] Lemańska K., Szymusiak H., Tyrakowska B., Zieliński R., Soffers A.E.M.F., Rietjens I.M.C.M. (2001). The influence of pH on antioxidant properties and the mechanism of antioxidant action of hydroxyflavones. Free Radic. Biol. Med..

[18] Boulebd H., Carmena-Bargueño M., Pérez-Sánchez H. (2023). Exploring the Antioxidant Properties of Caffeoylquinic and Feruloylquinic Acids: A Computational Study on Hydroperoxyl Radical Scavenging and Xanthine Oxidase Inhibition. Antioxidants.

[19] Galano A., Alvarez-Idaboy J.R. (2013). A computational methodology for accurate predictions of rate constants in solution: Application to the assessment of primary antioxidant activity. J. Comput. Chem..

[20] Galano A., Alvarez-Idaboy J.R. (2019). Computational strategies for predicting free radical scavengers’ protection against oxidative stress: Where are we and what might follow?. Int. J. Quantum Chem..

[21] Alberto M.E., Russo N., Grand A., Galano A. (2013). A physicochemical examination of the free radical scavenging activity of Trolox: Mechanism, kinetics and influence of the environment. Phys. Chem. Chem. Phys..

[22] Boulebd H. (2022). Radical scavenging behavior of butylated hydroxytoluene against oxygenated free radicals in physiological environments: Insights from DFT calculations. Int. J. Chem. Kinet..

[23] Boulebd H. (2022). Is cannabidiolic acid an overlooked natural antioxidant? Insights from quantum chemistry calculations. New J. Chem..

[24] Frisch M.J., Trucks G.W., Schlegel H.B., Scuseria G.E., Robb M.A., Cheeseman J.R., Scalmani G., Barone V., Mennucci B., Petersson G.A. (2009). Gaussian 09.

[25] Zhao Y., Truhlar D.G. (2008). The M06 suite of density functionals for main group thermochemistry, thermochemical kinetics, noncovalent interactions, excited states, and transition elements: Two new functionals and systematic testing of four M06-class functionals and 12 other functionals. Theor. Chem. Acc..

[26] Galano A., Alvarez-Idaboy J.R. (2014). Kinetics of radical-molecule reactions in aqueous solution: A benchmark study of the performance of density functional methods. J. Comput. Chem..

[27] Zhao Y., Truhlar D.G. (2008). How Well Can New-Generation Density Functionals Describe the Energetics of Bond-Dissociation Reactions Producing Radicals?. J. Phys. Chem. A.

[28] Marenich A.V., Cramer C.J., Truhlar D.G. (2009). Universal Solvation Model Based on Solute Electron Density and on a Continuum Model of the Solvent Defined by the Bulk Dielectric Constant and Atomic Surface Tensions. J. Phys. Chem. B.

[29] Lu T., Chen F. (2012). Multiwfn: A multifunctional wavefunction analyzer. J. Comput. Chem..

[30] Humphrey W., Dalke A., Schulten K. (1996). VMD: Visual molecular dynamics. J. Mol. Graph..

[31] Galano A., Pérez-González A., Castañeda-Arriaga R., Muñoz-Rugeles L., Mendoza-Sarmiento G., Romero-Silva A., Ibarra-Escutia A., Rebollar-Zepeda A.M., León-Carmona J.R., Hernández-Olivares M.A. (2016). Empirically Fitted Parameters for Calculating pKa Values with Small Deviations from Experiments Using a Simple Computational Strategy. J. Chem. Inf. Model..

[32] Evans M.G., Polanyi M. (1935). Some applications of the transition state method to the calculation of reaction velocities, especially in solution. Trans. Faraday Soc..

[33] Eyring H. (1935). The Activated Complex in Chemical Reactions. J. Chem. Phys..

[34] Truhlar D.G., Hase W.L., Hynes J.T. (1983). Current Status of Transition-State Theory. J. Phys. Chem. A.

[35] Pollak E., Pechukas P. (1978). Symmetry numbers, not statistical factors, should be used in absolute rate theory and in Broensted relations. J. Am. Chem. Soc..

[36] Fernández-Ramos A., Ellingson B.A., Meana-Pañeda R., Marques J.M., Truhlar D.G. (2007). Symmetry numbers and chemical reaction rates. Theor. Chem. Acc..

[37] Eckart C. (1930). The penetration of a potential barrier by electrons. Phys. Rev..

[38] Collins F.C., Kimball G.E. (1949). Diffusion-controlled reaction rates. J. Colloid Interface Sci..

[39] Corchado J.C., Coitino E.L., Chuang Y.-Y., Fast P.L., Truhlar D.G. (1998). Interpolated variational transition-state theory by mapping. J. Phys. Chem. A.

