# Supplementary material for: Hydroperoxyl Radical Scavenging Activity of Bromophenols from Marine Red Alga Polysiphonia urceolata: Mechanistic Insights, Kinetic Analysis, and Influence of Physiological Media
Source: Molecules. 2025 Apr 10;30(8):1697. doi: 10.3390/molecules30081697 (PMC12029166; doi:10.3390/molecules30081697)
Supplement: Supplementary file 1 [file molecules-30-01697-s001.zip › molecules-3555408-supplementary.pdf]

Hydroperoxyl Radical Scavenging Activity of Bromophenols from Marine Red Alga *Polysiphonia urceolata*: Mechanistic Insights, Kinetic Analysis, and Influence of Physiological Media

Houssem Boulebd[1,*]

[1]Laboratory of Synthesis of Molecules with Biological Interest, Department of Chemistry, Faculty of Exact Sciences, University of Frères Mentouri Constantine 1, Constantine 25017, Algeria

*Corresponding author: boulebd.houssem@umc.edu.dz

**Table of Contents**	Page
**Table S1**. The cartesian coordinates of the TSs of the reaction between BPs **1** and **2** and $HOO^{\bullet}$ radicals following *f*-HAT mechanism in physiological media at M06-2X/6-311++G(d,p) level.	S2-S23

Table S1. The cartesian coordinates of the TSs of the reaction between BPs **1** and **2** and $HOO^{\bullet}$ radicals following *f*-HAT mechanism in physiological media at M06-2X/6-311++G(d,p) level.

1-3O...H...OOH (PE)			
C	2.89154100	-0.07649200	-0.02801800
C	3.02406200	-1.34654800	0.52121700
C	1.87174600	-2.01392100	0.95542700
C	0.62736300	-1.42995100	0.83315700
C	0.50263100	-0.16629900	0.25062600
C	1.63989000	0.52860100	-0.18460700
C	-0.82371900	0.50516900	0.17500200
C	-0.93940600	1.82570800	0.69212600
C	0.29105300	2.49396900	1.26207700
O	1.22308600	2.89022400	0.26404600
C	1.45264900	1.91600600	-0.74858500
C	-1.95423900	-0.06896100	-0.39010100
C	-3.19972900	0.60313700	-0.40699700
C	-3.27721500	1.90536200	0.15128400
C	-2.14346100	2.51027600	0.67591100
O	-4.44681900	2.54735400	0.14374100
O	-4.29047700	0.11146600	-0.95480000
Br	-1.92702200	-1.76100200	-1.24200900
Br	4.49268100	0.80195500	-0.54312800
O	4.23300500	-1.92473300	0.65397800
O	2.10317900	-3.23832300	1.50421300
H	-0.24387200	-1.95374300	1.21093000
H	0.77428900	1.81517600	1.97457100
H	0.01229500	3.40544200	1.78981000

H	2.33237400	2.25298500	-1.29036200
H	0.60806000	1.90472200	-1.44589000
H	-2.21502700	3.51605500	1.07331400
H	-5.10152500	1.98503600	-0.29952400
H	-4.71947000	-0.65366400	-0.28432600
H	1.27584100	-3.67288000	1.74184600
O	-5.00150200	-1.34567700	0.76838700
O	-4.19631400	-0.78903000	1.70407400
H	-3.40893800	-1.36022700	1.73862800
H	4.11567000	-2.79883700	1.05057900
1-4O…H…OOH (PE)			
C	-2.95769400	-0.06830400	0.02466300
C	-3.18342800	1.12395000	0.70407800
C	-2.08112100	1.85576100	1.16646200
C	-0.79428800	1.41114200	0.94521000
C	-0.58031700	0.22403900	0.23940600
C	-1.66412600	-0.53410700	-0.22735500
C	0.79337900	-0.31222000	0.07393900
C	1.04067100	-1.65473800	0.46401000
C	-0.10159400	-2.49517400	0.98593700
O	-1.02721800	-2.87881600	-0.02434800
C	-1.37085000	-1.83818900	-0.93014000
C	1.85639300	0.43702000	-0.43675300
C	3.14538900	-0.08777100	-0.53174500
C	3.38905800	-1.42682100	-0.11305500
C	2.31031100	-2.18190300	0.38164600
O	4.57732000	-1.95349800	-0.25456500
O	4.20706400	0.59114200	-0.99853400
Br	1.60418200	2.18865200	-1.14767600

Br	-4.49050900	-1.04411500	-0.51901700
O	-4.43299300	1.56591400	0.93442700
O	-2.40442300	2.99191300	1.84181100
H	0.04164500	1.97054600	1.35095300
H	-0.62572400	-1.94987000	1.77926400
H	0.28452200	-3.42358200	1.40557600
H	-2.23383100	-2.19736100	-1.48479600
H	-0.55232800	-1.68194900	-1.64134000
H	2.50913600	-3.20473100	0.68155900
H	5.33464200	-1.40888600	0.37688400
H	-1.61269200	3.45490900	2.14024200
H	-4.38252400	2.40005800	1.42107700
O	5.92325400	-0.77253800	1.27236800
O	4.97392500	0.08160500	1.74048800
H	4.98618700	0.82357900	1.11152700
H	3.92628200	1.43663100	-1.37767200
1-10O…H…OOH (PE)			
C	-2.15679200	-0.25051400	-0.53673600
C	-2.55660200	1.02963500	-0.08080500
C	-1.56436400	1.85283500	0.52329300
C	-0.25249700	1.42067600	0.62212700
C	0.11551900	0.17554300	0.12123000
C	-0.84238600	-0.68432100	-0.47126900
C	1.50675900	-0.31971500	0.29312900
C	1.69025200	-1.59044400	0.86346100
C	0.48693500	-2.41924900	1.24645000
O	-0.20151000	-2.96125700	0.12359600
C	-0.38191600	-2.04168200	-0.94639800
C	2.64224500	0.41259900	-0.08757200

C	3.92533000	-0.07886800	0.12842600
C	4.07797400	-1.33796900	0.72139800
C	2.97257800	-2.09077000	1.07089500
O	5.36931500	-1.72987100	0.89095400
O	5.00953200	0.63067700	-0.24192000
Br	2.51890600	2.06199900	-1.01621900
Br	-3.53310400	-1.35162500	-1.22914600
O	-3.78343700	1.46362600	-0.19585000
O	-1.99974900	3.03001900	0.99073200
H	0.48131200	2.05039100	1.11297500
H	-0.20514900	-1.81302900	1.84306300
H	0.79330100	-3.27475000	1.84800100
H	-1.10777700	-2.49799100	-1.61414300
H	0.55835800	-1.92233200	-1.49469500
H	3.10725100	-3.07370600	1.51252500
H	5.41418200	-2.62148700	1.25622200
H	5.80120500	0.11514500	-0.03606100
H	-1.27906200	3.50499600	1.42429900
O	-4.52917200	1.26593500	2.03441600
O	-3.39208400	0.66942100	2.47804500
H	-3.59530100	-0.28194700	2.47820700
H	-4.23277000	1.53668200	0.81183500
1-11O…H…OOH (PE)			
C	-2.18710500	-0.48097000	-0.50071800
C	-2.63482800	0.80860400	-0.21583000
C	-1.70201700	1.77876400	0.25412100
C	-0.36075400	1.39917400	0.42857100
C	0.07130700	0.12709700	0.10886700
C	-0.84972000	-0.84216100	-0.36210400

C	1.48324000	-0.28489900	0.33736800
C	1.71824100	-1.45086400	1.08179700
C	0.54564800	-2.25274700	1.59099200
O	-0.12779800	-2.97567000	0.55984700
C	-0.33352500	-2.23578200	-0.63431300
C	2.58426500	0.41794500	-0.17224500
C	3.88695400	0.00132900	0.08270400
C	4.09247700	-1.15281900	0.84694000
C	3.01972400	-1.87859500	1.32949200
O	5.40004500	-1.47799500	1.03894400
O	4.93984000	0.68160600	-0.41224700
Br	2.38721200	1.92008500	-1.31445000
Br	-3.50127500	-1.73073200	-1.09063600
O	-3.91024700	1.21176500	-0.31857900
O	-2.09687000	3.00277200	0.48888100
H	0.32060300	2.14206100	0.82463100
H	-0.16794300	-1.59117700	2.09536000
H	0.88086300	-3.00317700	2.30644900
H	-1.03149700	-2.81762400	-1.23105200
H	0.60453100	-2.15354900	-1.19367500
H	3.19555100	-2.78292000	1.90391500
H	5.48155400	-2.29604800	1.54322600
H	5.75187300	0.23058700	-0.14413200
H	-2.82640100	3.02075900	1.35359000
H	-4.45084900	0.52180700	-0.72948700
O	-3.46025200	2.78338200	2.39256200
O	-3.33368000	1.43830400	2.54789600
H	-4.05782300	1.06415400	2.01690800

2-3O…H…OOH (PE)

C	2.90766900	-0.15156800	0.07442100
C	3.19122900	1.16135700	-0.28607000
C	2.15956700	1.94249200	-0.81660500
C	0.87791800	1.44335000	-0.92205400
C	0.58810800	0.14016100	-0.50357100
C	1.62711500	-0.69441400	-0.05581700
C	-0.78460000	-0.42063700	-0.57941100
C	-0.90774900	-1.81097100	-0.86160800
C	0.35531000	-2.62295200	-0.91423600
C	1.31734300	-2.15090800	0.17630700
C	-1.96289000	0.28800100	-0.36473500
C	-3.23442200	-0.30872700	-0.53046800
C	-3.30234200	-1.67805000	-0.88856600
C	-2.13993700	-2.42043300	-1.02619700
O	-4.49837200	-2.24960100	-1.05258200
O	-4.37800700	0.33677400	-0.38917200
Br	-2.02356100	2.05443500	0.33917100
Br	4.35077800	-1.18238500	0.75376900
O	4.43270600	1.66806500	-0.15683400
O	2.53848700	3.19593000	-1.19970200
H	0.11051500	2.06933600	-1.35843500
H	-2.20789400	-3.48004700	-1.24311500
H	-5.17916200	-1.57200600	-0.91612000
H	-4.61711500	0.42933200	0.67093800
H	1.78170800	3.70122200	-1.51842800
H	0.11646900	-3.68029100	-0.79369300
H	0.83513800	-2.49699000	-1.89240700
H	0.86155700	-2.29513400	1.16285300
H	2.22921600	-2.74436300	0.14797800

H	4.42494600	2.58186600	-0.47271300
O	-4.64288000	0.23376300	1.97134000
O	-3.59284900	-0.59938000	2.15278100
H	-2.83454300	-0.01931400	2.34523100
2-4O…H…OOH (PE)			
C	-3.00822200	-0.22488400	0.07566800
C	-3.25376600	1.05403700	0.56619100
C	-2.16264100	1.84720000	0.94231000
C	-0.87202800	1.39695500	0.77115400
C	-0.63075400	0.13101500	0.22074600
C	-1.71222000	-0.72180200	-0.07241900
C	0.73969800	-0.37701600	0.00142900
C	0.95561000	-1.77051800	0.18008600
C	-0.24966400	-2.64158000	0.40800700
C	-1.40950300	-2.14750900	-0.45549100
C	1.83965200	0.40634900	-0.38006500
C	3.12476900	-0.12652900	-0.47184600
C	3.33578700	-1.50889900	-0.20631300
C	2.22123700	-2.30390800	0.10783100
O	4.52447200	-2.03640000	-0.35627800
O	4.21986600	0.58243800	-0.80953900
Br	1.67066700	2.21344700	-0.98467000
Br	-4.52486300	-1.26793400	-0.38530000
O	-4.50871800	1.51458100	0.70930900
O	-2.50068300	3.05550600	1.47543200
H	-0.05404700	2.02159300	1.10696900
H	-0.54641500	-2.60473100	1.46272600
H	-0.00002000	-3.67614300	0.16839500
H	-2.28411600	-2.77719100	-0.30407800

H	-1.13865600	-2.20857100	-1.51601400
H	2.38626600	-3.36506000	0.25742100
H	5.26534400	-1.55840300	0.32043000
H	-1.71507600	3.58318500	1.66038300
H	-4.46994700	2.40948800	1.07427700
O	5.86442400	-1.01799100	1.29912200
O	4.91160700	-0.22598000	1.85943500
H	4.89040500	0.56314300	1.29097900
H	3.96764300	1.46505400	-1.11610800
2-11O…H…OOH (PE)			
C	2.19091700	0.46026700	-0.42235300
C	2.56370700	-0.88807900	-0.20291600
C	1.55888400	-1.77239200	0.26948400
C	0.25283700	-1.33900400	0.42215100
C	-0.10364100	-0.02658800	0.11555700
C	0.89676200	0.91579900	-0.24970100
C	-1.50210900	0.45004400	0.22954100
C	-1.69589300	1.77918700	0.64360000
C	-0.49141600	2.67234700	0.77656000
C	0.49390500	2.36342100	-0.34984100
C	-2.64699700	-0.32474600	-0.04975100
C	-3.92662300	0.16401200	0.18345300
C	-4.07778600	1.46519500	0.67823400
C	-2.97463900	2.27268800	0.87733300
O	-5.36628700	1.85022500	0.87863000
O	-5.01770000	-0.58301000	-0.09042000
Br	-2.57792000	-2.01199300	-0.92410700
Br	3.58202600	1.62197600	-0.97976100
O	3.78092800	-1.32376600	-0.41138500

O	1.96568200	-3.02255200	0.54103700
H	-0.48435200	-2.03477100	0.80127400
H	-3.10889300	3.29873500	1.20555800
H	-5.40990900	2.76702000	1.17535800
H	-5.80579000	-0.05735800	0.10147700
H	1.23569300	-3.53815500	0.90574200
O	4.55553100	-1.58253500	1.81592300
O	3.42656900	-1.08399400	2.38112700
H	3.63605300	-0.15438300	2.57547300
H	4.22527700	-1.60999900	0.53914300
H	-0.80480300	3.71679000	0.73707100
H	0.00107900	2.51016500	1.74266600
H	0.01894700	2.55677300	-1.31895900
H	1.36776300	3.00795600	-0.27682800
2-12O…H…OOH (PE)			
C	-2.23514300	-0.61665700	-0.38837800
C	-2.63300800	0.71522200	-0.27169100
C	-1.66255300	1.69090100	0.08623600
C	-0.33061900	1.28648100	0.26940600
C	0.06444200	-0.02374100	0.07492000
C	-0.91804800	-1.01732000	-0.19207900
C	1.47519300	-0.46102100	0.23313300
C	1.69587900	-1.73651100	0.77581400
C	0.50790100	-2.63643500	0.99342200
C	-0.48433100	-2.45590900	-0.15628100
C	2.60021200	0.30931300	-0.11847200
C	3.89016600	-0.12504500	0.16582900
C	4.06950800	-1.36687400	0.78540400
C	2.98441700	-2.17707500	1.05920100

O	5.36704800	-1.69863000	1.02840700
O	4.96432800	0.61918000	-0.17461200
Br	2.49433000	1.91068700	-1.13708200
Br	-3.59919800	-1.88000500	-0.82024300
O	-3.89436500	1.14962600	-0.42505500
O	-2.00764400	2.95035600	0.19703900
H	0.36939100	2.05154600	0.57380000
H	3.13945700	-3.16259700	1.48807100
H	5.42881800	-2.57677700	1.42220900
H	5.76294200	0.14530400	0.09305400
H	-2.74061100	3.08134600	1.02623100
H	-4.45877300	0.43529400	-0.75361200
O	-3.40645600	2.98252000	2.09735600
O	-3.32713500	1.65891000	2.40265400
H	-4.04904900	1.25129900	1.89446900
H	0.83796600	-3.67487000	1.04789900
H	0.01041100	-2.39547800	1.93997800
H	-0.00129400	-2.71964400	-1.10510300
H	-1.33916200	-3.11698500	-0.02572700
1-3O…H…OOH (W)			
C	-2.92101100	0.02038600	0.00166100
C	-3.09861500	1.26164400	0.60249400
C	-1.96877900	1.95883200	1.05057400
C	-0.70402200	1.42955900	0.88766500
C	-0.53652000	0.19695800	0.25512800
C	-1.64926900	-0.52996200	-0.19064100
C	0.81603900	-0.41004800	0.13552100
C	0.99575500	-1.74148500	0.60638900
C	-0.19019100	-2.48014600	1.17953100

O	-1.12834600	-2.88514100	0.18029300
C	-1.41228200	-1.88705800	-0.80332600
C	1.90828700	0.23911300	-0.42027000
C	3.17971800	-0.37680200	-0.48933100
C	3.31826300	-1.69978500	0.00806100
C	2.22380500	-2.37124800	0.54116800
O	4.50917100	-2.31187800	-0.04951600
O	4.23826800	0.20661100	-1.02299800
Br	1.75701300	1.94524100	-1.23296400
Br	-4.49103300	-0.90655400	-0.53375900
O	-4.33705300	1.78561100	0.76820600
O	-2.22590500	3.15808200	1.64986300
H	0.14629600	1.97771300	1.27737300
H	-0.69246200	-1.84850900	1.91976600
H	0.13966400	-3.39595700	1.66748900
H	-2.28183700	-2.24681800	-1.34651100
H	-0.57539100	-1.82340300	-1.50545600
H	2.34844700	-3.38546400	0.90208100
H	5.15404700	-1.73001200	-0.48195100
H	4.90242700	0.53914400	-0.21422800
H	-1.40205200	3.58408400	1.92060500
O	5.42423000	0.77940000	0.95784100
O	4.57974200	0.12549800	1.79045800
H	3.95797900	0.79865400	2.12112600
H	-4.25431700	2.65129200	1.19407500
1-4O…H…OOH (W)			
C	-2.92559300	-0.07044700	0.02240100
C	-3.15679900	1.10621900	0.72706700
C	-2.05644000	1.84391000	1.18598300

C	-0.76801700	1.41244100	0.94224400
C	-0.55019500	0.23805900	0.21874800
C	-1.63158600	-0.52102600	-0.25230100
C	0.82504900	-0.29073100	0.03982800
C	1.07390200	-1.63893000	0.41355100
C	-0.06083000	-2.48625300	0.93552100
O	-0.98927000	-2.86625800	-0.08339000
C	-1.33622900	-1.80939600	-0.97887800
C	1.87873300	0.46680500	-0.47476400
C	3.16783100	-0.05887200	-0.58869800
C	3.40947900	-1.39861200	-0.17286700
C	2.34261800	-2.16599000	0.31859600
O	4.62502500	-1.90039500	-0.29164200
O	4.22710000	0.59612900	-1.07767400
Br	1.61253000	2.22518900	-1.15349500
Br	-4.45218300	-1.05749000	-0.52679800
O	-4.41734100	1.52633800	0.98386900
O	-2.36784300	2.97276100	1.88532000
H	0.05847800	1.98153100	1.35316700
H	-0.59084800	-1.94908000	1.72865700
H	0.32890800	-3.41659600	1.34568400
H	-2.19619100	-2.16370800	-1.54082200
H	-0.51531100	-1.64483300	-1.68329300
H	2.54440600	-3.18936900	0.61477900
H	5.28533100	-1.37726300	0.42223800
H	-1.56505700	3.42865200	2.17089800
H	-4.37731800	2.34977800	1.49198800
O	5.70924600	-0.70662100	1.44574900
O	4.58292100	-0.06241700	1.84834800

H	4.18250700	-0.63330200	2.52800100
H	4.00267600	1.50535400	-1.32427300
1-10O…H…OOH (W)			
C	-2.16817300	-0.30839800	-0.52214900
C	-2.57852100	0.97563900	-0.09105900
C	-1.58979200	1.83677200	0.46152200
C	-0.27160300	1.42481800	0.56945100
C	0.10443700	0.16757000	0.11396500
C	-0.84838200	-0.72002600	-0.45188900
C	1.49847100	-0.31186100	0.29707300
C	1.69335200	-1.56285400	0.90607300
C	0.50060300	-2.39436100	1.31006400
O	-0.17731100	-2.97477600	0.19016100
C	-0.36864800	-2.07772900	-0.90304100
C	2.62839600	0.41578900	-0.10686900
C	3.91577200	-0.05780400	0.12358600
C	4.07989800	-1.29240300	0.76382700
C	2.97884700	-2.04255800	1.13560700
O	5.37298300	-1.67748100	0.95891500
O	4.99977400	0.65143000	-0.27699000
Br	2.48504900	2.03638300	-1.08530400
Br	-3.51999900	-1.43689900	-1.22326900
O	-3.82121300	1.39485500	-0.19514800
O	-2.02819300	3.03663100	0.87602600
H	0.44808500	2.08873100	1.03389800
H	-0.20765400	-1.78604000	1.88245100
H	0.81733900	-3.23150900	1.93004600
H	-1.08420900	-2.55887900	-1.56404100
H	0.57232700	-1.95738800	-1.44762100

H	3.12905700	-3.00568200	1.61295100
H	5.40821700	-2.53749200	1.39761500
H	5.79801600	0.15560700	-0.04329700
H	-1.30017200	3.54525900	1.26046900
O	-4.44829500	1.45596000	2.09668800
O	-3.28717100	0.92104400	2.55209100
H	-3.46731000	-0.02771300	2.67583700
H	-4.22301200	1.55631400	0.81082200
1-11O…H…OOH (W)			
C	-2.24013900	-0.41425000	-0.50902000
C	-2.65398200	0.88464700	-0.20813200
C	-1.68563600	1.82410600	0.25126600
C	-0.35157300	1.42106500	0.40209400
C	0.04500900	0.14050800	0.07087100
C	-0.90850400	-0.80171600	-0.39358500
C	1.44545300	-0.30884900	0.29082000
C	1.65542300	-1.48886500	1.02115300
C	0.46989900	-2.27182700	1.52671200
O	-0.22609100	-2.96792100	0.48378000
C	-0.42585600	-2.19964400	-0.69894900
C	2.56426800	0.37797800	-0.20065100
C	3.85801100	-0.06294300	0.06025200
C	4.03829100	-1.23080100	0.81105200
C	2.94645600	-1.94291700	1.27334200
O	5.33736600	-1.59139100	1.02190800
O	4.93038600	0.61515600	-0.41876700
Br	2.39805700	1.89959000	-1.32535500
Br	-3.58634300	-1.63942000	-1.06923000
O	-3.90860800	1.33836600	-0.29181200

O	-2.07120700	3.05196700	0.53872400
H	0.34893500	2.14388800	0.80181600
H	-0.23191000	-1.60481600	2.03893400
H	0.79436700	-3.03960700	2.22727300
H	-1.13928900	-2.75513000	-1.30182100
H	0.51063400	-2.12830900	-1.26013400
H	3.10814900	-2.85478500	1.83925600
H	5.38458000	-2.40862000	1.53494400
H	5.73809800	0.16069000	-0.13890400
H	-2.69545000	3.00649200	1.46267100
H	-4.52509700	0.64315700	-0.56411600
O	-3.20432700	2.62007700	2.56765600
O	-2.88312000	1.29996300	2.58773200
H	-2.04089900	1.24241500	3.07342000
2-3O…H…OOH (W)			
C	-2.97358600	-0.11225800	0.06519200
C	-3.14468800	1.20592400	0.47702900
C	-2.00953500	1.95158800	0.81259300
C	-0.74787800	1.41059800	0.67431500
C	-0.58182900	0.10361000	0.20364800
C	-1.71011100	-0.69696100	-0.04544200
C	0.76265000	-0.49799600	0.02842600
C	0.89203100	-1.89156100	0.29779400
C	-0.36532400	-2.66724700	0.56857300
C	-1.49037200	-2.15939000	-0.33443100
C	1.89969600	0.17831300	-0.40268000
C	3.16420000	-0.44986600	-0.44518900
C	3.25886100	-1.81538600	-0.07840400
C	2.11739400	-2.52983300	0.25881400

O	4.45139000	-2.43196500	-0.10432900
O	4.26706700	0.16375600	-0.85834800
Br	1.85292100	1.93589500	-1.12532600
Br	-4.54452400	-1.09751700	-0.34999500
O	-4.38115000	1.75123600	0.58323900
O	-2.25826000	3.21649800	1.27380600
H	0.10105200	2.01496000	0.96634500
H	-0.66204100	-2.53535600	1.61593900
H	-0.18139000	-3.72937300	0.40433900
H	-2.39843800	-2.72893200	-0.14740000
H	-1.21821600	-2.30726400	-1.38563500
H	2.19711300	-3.58928300	0.47264600
H	5.13192300	-1.80202200	-0.39066100
H	4.83289300	0.48033700	0.00097600
H	-1.43050600	3.67228200	1.47447600
O	5.25534000	0.66934600	1.26492800
O	4.41328300	-0.13190400	1.95626400
H	3.71568300	0.45082100	2.30713600
H	-4.29237200	2.65766800	0.91217800
2-4O…H…OOH (W)			
C	-2.97156700	-0.21866000	0.06592100
C	-3.21577900	1.04839000	0.58937700
C	-2.12235000	1.83610800	0.97110400
C	-0.83357700	1.39065100	0.76820500
C	-0.59573900	0.13738200	0.19059200
C	-1.67815600	-0.71249900	-0.10825700
C	0.77445300	-0.36492100	-0.04615000
C	0.98836500	-1.76224900	0.11358700
C	-0.21438000	-2.63754900	0.33132600

C	-1.37709900	-2.13018400	-0.51959700
C	1.86810800	0.42562400	-0.42761500
C	3.15411100	-0.10822600	-0.52983500
C	3.35928600	-1.48777600	-0.25759400
C	2.25495700	-2.29493700	0.03840000
O	4.58092700	-1.99311600	-0.35911700
O	4.24861000	0.57702100	-0.89653800
Br	1.69286400	2.24032500	-0.99706400
Br	-4.48837100	-1.26236700	-0.40018100
O	-4.47896300	1.50098100	0.75656000
O	-2.44162100	3.03760400	1.54120300
H	-0.01937300	2.01764600	1.10698500
H	-0.50779900	-2.61724000	1.38726500
H	0.03707200	-3.66699500	0.07390300
H	-2.24874300	-2.76517200	-0.37553800
H	-1.10810500	-2.17136100	-1.58116400
H	2.41987800	-3.35670400	0.18366300
H	5.20120100	-1.54222200	0.39998200
H	-1.64165200	3.51945300	1.78912000
H	-4.44410300	2.38398600	1.15319200
O	5.59914300	-0.95299900	1.53930900
O	4.45912600	-0.33431200	1.93922200
H	4.02313700	-0.95329700	2.55120200
H	4.04963400	1.51502400	-1.02693600
2-11O…H…OOH (W)			
C	2.20073400	0.48964100	-0.40727000
C	2.57746300	-0.85462900	-0.18604400
C	1.57364500	-1.75513200	0.25058200
C	0.26199300	-1.33562600	0.39363600

C	-0.09625000	-0.02070100	0.10703900
C	0.90252900	0.93355000	-0.23898800
C	-1.49463200	0.44915600	0.22465000
C	-1.69421900	1.76810800	0.66796200
C	-0.49559800	2.66713400	0.80807900
C	0.48869800	2.37820900	-0.32481000
C	-2.63626400	-0.32653000	-0.06503800
C	-3.91685500	0.14468500	0.19348200
C	-4.07391800	1.43227700	0.72454300
C	-2.97399700	2.24461000	0.92714200
O	-5.36112600	1.81178100	0.95937900
O	-5.01023800	-0.61134400	-0.08948300
Br	-2.55149100	-1.98300900	-0.99718000
Br	3.57061300	1.66364900	-0.99320900
O	3.81236000	-1.28703800	-0.37302400
O	1.98136600	-3.01588000	0.50067900
H	-0.46554300	-2.05145800	0.75283100
H	-3.12047500	3.25992500	1.28124000
H	-5.38997100	2.71477100	1.30146100
H	-5.80334500	-0.10238400	0.13147100
H	1.23840000	-3.54979300	0.81406700
O	4.48015200	-1.69577200	1.89364700
O	3.32954500	-1.23704500	2.44105200
H	3.50812100	-0.31329700	2.69166100
H	4.21930700	-1.59864900	0.56824700
H	-0.81767400	3.70874600	0.78095800
H	0.00100600	2.49690400	1.77018900
H	0.00836900	2.57646400	-1.28997900
H	1.35611100	3.03024700	-0.24631700

2-12O…H…OOH (W)			
C	-2.25602300	-0.60049200	-0.38931000
C	-2.64258300	0.73706100	-0.27941700
C	-1.66103900	1.70086300	0.07643600
C	-0.33363400	1.29689800	0.26069000
C	0.05062500	-0.01772900	0.07304700
C	-0.94190600	-1.00612900	-0.18665300
C	1.45723700	-0.46383100	0.22692200
C	1.67215700	-1.73959800	0.77267200
C	0.48026400	-2.63142900	0.99620400
C	-0.51609400	-2.44617500	-0.14852700
C	2.58720400	0.29834000	-0.12787300
C	3.87498700	-0.14175200	0.15523500
C	4.04897000	-1.38389400	0.77768600
C	2.95821800	-2.18648800	1.05496200
O	5.34433500	-1.73289300	1.02573500
O	4.95946000	0.60216200	-0.18931800
Br	2.48244700	1.89550300	-1.15785000
Br	-3.61635600	-1.86085400	-0.82909800
O	-3.88843900	1.20208600	-0.44813600
O	-2.01382300	2.97274100	0.18889700
H	0.37075300	2.05848900	0.56363900
H	3.11578000	-3.17037800	1.48525600
H	5.38634000	-2.60874800	1.43077000
H	5.75727000	0.12488700	0.08017300
H	-2.69146500	3.08514400	1.03535200
H	-4.50999400	0.48981600	-0.65854600
O	-3.33867600	2.93756000	2.16831800
O	-3.06032800	1.65042800	2.50185600

H	-3.81489100	1.13042800	2.17279500
H	0.80615100	-3.67064500	1.04946200
H	-0.01139100	-2.38657900	1.94441300
H	-0.03922600	-2.71310700	-1.09935000
H	-1.37302200	-3.10257300	-0.01203800
1-(11O⁻)-3O…H…OOH (W)			
C	2.92847000	-0.05310400	0.01386500
C	3.08915300	-1.27194300	0.63898700
C	1.96427100	-2.01532100	1.12055900
C	0.70111900	-1.44200600	0.91457900
C	0.54941400	-0.21960900	0.25467900
C	1.65672900	0.50642500	-0.20480700
C	-0.80054100	0.39593200	0.12798700
C	-0.97360600	1.73296600	0.58927400
C	0.21714400	2.47350400	1.15146100
O	1.14673000	2.87429100	0.14400900
C	1.42934200	1.85482100	-0.82781300
C	-1.90159700	-0.24813100	-0.41757600
C	-3.16934900	0.37482300	-0.48530600
C	-3.29936700	1.70110000	0.00297800
C	-2.19873100	2.36992800	0.52511100
O	-4.48919600	2.31952500	-0.05351400
O	-4.23624900	-0.20491200	-1.01217800
Br	-1.76730300	-1.96140800	-1.21999500
Br	4.51060800	0.86515500	-0.53581200
O	4.31202800	-1.83521000	0.84958000
O	2.19471100	-3.14682500	1.71642300
H	-0.16775700	-1.96755500	1.29418700
H	0.72320800	1.84317300	1.89021900

H	-0.11009200	3.39102800	1.63872200
H	2.30231500	2.20636600	-1.37180100
H	0.59141500	1.80088500	-1.52979300
H	-2.31588800	3.38754800	0.87915600
H	-5.13730900	1.73647700	-0.47916800
H	-4.89020400	-0.53511600	-0.20413500
O	-5.41044000	-0.77116700	0.98436700
O	-4.56032100	-0.11062900	1.80551900
H	-3.93090400	-0.77901800	2.13154400
H	4.10673700	-2.67186000	1.30679100
1-(11O⁻)-4O…H…OOH (W)			
C	-2.93481900	-0.04556900	0.03915800
C	-3.14946600	1.11303400	0.75757400
C	-2.05785800	1.89204000	1.26127900
C	-0.76927900	1.41802600	0.97902700
C	-0.56504000	0.25301900	0.23218900
C	-1.64041300	-0.50596700	-0.25342100
C	0.80827900	-0.27646000	0.03971000
C	1.05822600	-1.63260000	0.38852400
C	-0.07375400	-2.48906000	0.90291500
O	-1.00075800	-2.86184600	-0.11828200
C	-1.35453100	-1.78280200	-0.99432900
C	1.86832200	0.48489000	-0.45997000
C	3.15525300	-0.04221800	-0.58527000
C	3.39352000	-1.38936200	-0.19772300
C	2.32544000	-2.16176600	0.28060900
O	4.60922400	-1.89606200	-0.33030100
O	4.21700100	0.62153300	-1.06278600
Br	1.61447900	2.25600800	-1.11228200

Br	-4.47305900	-1.02226800	-0.52796000
O	-4.39578200	1.57743900	1.04437900
O	-2.34103900	2.95891800	1.94416700
H	0.07486000	1.96607000	1.38189800
H	-0.60308100	-1.95929100	1.70141600
H	0.32012500	-3.42176000	1.30460600
H	-2.22002300	-2.12952100	-1.55329000
H	-0.53635900	-1.62269900	-1.70320700
H	2.52503900	-3.19085900	0.55837600
H	5.26728500	-1.40082600	0.38954400
H	-4.23017800	2.39178000	1.55568500
O	5.70003100	-0.75027700	1.44471600
O	4.57603800	-0.10793800	1.85446700
H	4.16350500	-0.69307100	2.51444000
H	3.98813600	1.53390500	-1.29350900
